# CHIME-GP trial of online education for prescribing, pathology and imaging ordering in general practice – how did it bring about behaviour change?

**DOI:** 10.1186/s12913-023-10374-1

**Published:** 2023-12-02

**Authors:** Christine Metusela, Judy Mullan, Conrad Kobel, Joel Rhee, Marijka Batterham, Stephen Barnett, Andrew Bonney

**Affiliations:** 1 Graduate School of Medicine, University of Wollongong, Wollongong, Australia; 2 Faculty of Science, Medicine and Health, University of Wollongong, Wollongong, Australia; 3 School of Population Health, University of New South Wales, Sydney, Australia; 4 School of Mathematics and Applied Statistics, University of Wollongong, Wollongong, Australia

**Keywords:** General practice, Primary care, Electronic health record, Rational prescribing, Test ordering

## Abstract

**Background:**

There is a need for scalable clinician education in rational medication prescribing and rational ordering of pathology and imaging to help improve patient safety and enable more efficient utilisation of healthcare resources. Our wider study evaluated the effectiveness of a multifaceted education intervention for general practitioners (GPs) in rational prescribing and ordering of pathology and imaging tests, in the context of Australia’s online patient-controlled health record system, My Health Record (MHR), and found evidence for measurable behaviour change in pathology ordering among participants who completed the educational activities. This current study explored the mechanisms of behaviour change brought about by the intervention, with a view to informing the development of similar interventions in the future.

**Methods:**

This mixed methods investigation used self-reported questionnaires at baseline and post-education on MHR use and rational prescribing and test ordering. These were analysed using multi-level ordinal logistic regression models. Semi-structured interviews pre- and post-intervention were also conducted and were analysed thematically using the COM-B framework.

**Results:**

Of the 106 GPs recruited into the study, 60 completed baseline and 37 completed post-education questionnaires. Nineteen participants were interviewed at baseline and completion. Analysis of questionnaires demonstrated a significant increase in confidence using MHR and in self-reported frequency of MHR use, post-education compared with baseline. There were also similar improvements in confidence across the cohort pre-post education in deprescribing, frequency of review of pathology ordering regimens and evidence-based imaging. The qualitative findings showed an increase in GPs’ perceived capability with, and the use of MHR, at post-education compared with baseline. Participants saw the education as an opportunity for learning, for reinforcing what they already knew, and for motivating change of behaviour in increasing their utilisation of MHR, and ordering fewer unnecessary tests and prescriptions.

**Conclusions:**

Our education intervention appeared to provide its effects through providing opportunity, increasing capability and enhancing motivation to increase MHR knowledge and usage, as well as rational prescribing and test ordering behaviour. There were overlapping effects of skills acquisition and confidence across intervention arms, which may have contributed to wider changes in behaviour than the specific topic area addressed in the education.

**Trial registration:**

Australian New Zealand Clinical Trials Registry (ACTRN12620000010998) (09/01/2020).

**Supplementary Information:**

The online version contains supplementary material available at 10.1186/s12913-023-10374-1.

## Introduction

The over prescribing and unnecessary prescribing of prescription medicines and test ordering is a major concern for health systems internationally [1–3]. The rational ordering of prescription medications, and pathology and imaging has significant implications for patient safety and efficient utilisation of healthcare resources and budgets [4]. Unnecessary ordering of prescriptions and tests drives up health system costs, creates system-wide inefficiencies, and places patients at increased risk of harm [5–7]. Unsafe medication practices are a leading cause of injury and avoidable harm in health care systems across the world with a global annual cost estimated at USD$42 billion [8]. In Australia, an estimated 250,000 hospital admissions are medication related, with an annual cost of AUD$1.4 billion to the healthcare system [9]. Likewise, research from the UK suggests that 25% of pathology requests are either unnecessary or inappropriate [10] and globally that 50% of imaging tests are of low value [11].

There is international momentum to reduce unnecessary medicalisation in order to reduce these risks, supported by peak medical bodies in various countries, including the UK, USA, and Australia [1, 5, 12]. Under the umbrella of ‘Choosing Wisely’, evidence based, discipline, and country specific recommendations for reducing key unnecessary prescriptions and tests have been developed and made publicly available [13]. However, to be most effective in changing clinical behaviour, guidelines and recommendations require implementation strategies [14].

Many interventions have been trialled in attempts to change and rationalise prescribing and test ordering patterns, however often the size of the effect of these trials has been limited (i.e. less than 25% change) [3, 15, 16]. This may have been due to difficulties implementing the trial intervention in practices or failing to address the underlying drivers of variability in physician behaviour [3, 15]. Interventions that include the use of guidelines, audits, reflective practice, workshops, and academic detailing have shown some positive changes [2, 17–20]. The most effective interventions include those that adopt a multifaceted or multicomponent approach with blended learning, in particular practitioner education and feedback combined with systems change [3, 17]. GP alerting systems combined with practitioner education, including online tools and feedback have been shown to be beneficial in changing test ordering practices [21]. One such study that used a targeted reminder intervention was effective in reducing low-back pain imaging referrals by 22.5% [22]. Education, feedback and system change have proven the most successful for reductions in pathology ordering [3], and clinical decision support technologies and drug usage advice have proved somewhat effective for rational prescribing [23]. However, it should be noted that the real-world effects of electronic health record clinical alerts can be blunted by ‘alert fatigue’, with physicians overriding alerts due to perceived lack of usefulness or desensitization [24]. In addition, methods that have enabled practitioners to compare ordering and prescribing statistics have demonstrated to be effective [25].

Educational interventions on rational prescribing and test ordering in general practice have the potential for significant savings to the Medicare Benefits Schedule (MBS) and the Pharmaceutical Benefits Scheme (PBS). However, there is a paucity of robust randomised controlled trials which include scalable education interventions to reduce unnecessary medication prescribing [26] and low value diagnostic testing [3, 22]. In addition, exploring and influencing medical practitioner habits can be challenging and requires a pragmatic approach [27].

The opportunity to deploy a multifaceted education intervention facilitated by a digital health record system has the potential to augment educational impact and to help change behaviour. Importantly, as an implementation strategy it can also potentially address barriers of uptake at a systems level [14]. My Health Record (MHR), established in 2012, is a national patient-controlled digital health record system administered by the Australian Digital Health Agency (ADHA). All Australians have a MHR, unless they opted-out prior to 31 January 2019. MHR is a secure online summary of patients’ health information. As an electronic health record (EHR), MHR helps in facilitating person-centred care such as patient engagement and shared decision-making [28]. Patients can control what goes into their MHR and who is allowed to access it. They can also choose to permanently delete their record. Key aims of the MHR are to improve medication safety and reduce unnecessary test duplication [29].

MHR provides a new context for quality and safety improvement interventions in healthcare. It is timely therefore to investigate an appropriately designed, online education intervention, coupled with MHR. Our wider study evaluated the effectiveness of a multifaceted education package for GPs regarding rational prescribing and ordering of pathology and radiology tests, in the context of the MHR system. We found evidence for measurable behaviour change in pathology ordering among those completing the educational activities. There was also evidence of overlapping effect of the education across study arms, particularly pathology and imaging arms [30]. This present study focused on the self-reported pre- and post-education questionnaires and the qualitative pre- and post-interviews to explore behaviour change in further depth, including the mechanisms by which behaviour change were brought about. The aim of this paper was to explore the mechanisms of the intervention with a view to informing the development of similar interventions in the future.

## Method

The present study was part of a pragmatic cluster-randomised three arm parallel trial, with participants randomised into prescribing, imaging and pathology arms. An educational intervention package was delivered over a period of three months that included three webinars (tailored for each arm), an asynchronous online education module, an audit and a pre- and post-intervention questionnaire. The methods of the trial are reported in further detail elsewhere [31, 32].

Following ethics approval through the University of Wollongong and Illawarra Shoalhaven Local Health District Health and Medical Human Research Ethics Committee, the trial was registered with the Australian New Zealand Clinical Trials Registry (ACTRN12620000010998). Medcast Pty Ltd, PenCS, the Australian Digital Health Agency (ADHA) the University of Wollongong (UOW), the Royal Australian College of General Practitioners (RACGP) and Primary Health Networks across Australia disseminated information about the trial to practices and GPs involved in their pre-existing networks.

During the consent process of the trial, GPs were also asked if they were willing to be contacted regarding participation in pre-and post-intervention interviews. A maximum diversity sample of these consenting GPs, diversified by practice size, remoteness [33] and socio-economic index for areas (SEIFA) [34], and age and sex of participants, were then contacted for interviews. Nineteen GPs participated in both a pre- and a post-interview. Interviews were conducted over the telephone by an experienced qualitative researcher (CM) and were recorded and transcribed verbatim. The pre- and post-interview guides are provided in Additional File 1.

### Quantitative questionnaire analysis

All participants were invited to complete pre- and post-education questionnaires, which were identical across all three study arms. Questions included Likert-type response items for confidence in MHR use, rational prescribing and test ordering. Categorical items recorded responses for self-assessed frequency of MHR use and evidence-based prescribing, pathology, and imaging clinical activities. The pre- and post-education questionnaires are provided in Additional File 2. As the MHR education component was similar in all three arms, MHR-related items were assessed for changes pre- and post-education across all study arms. Between-arm differences in changes in topic specific items pre-and post-intervention were analysed using multi-level ordinal logistic regression models. The GP participant ID was included as a random effect to account for repeated measures within individuals in regression models. All available data for items were used in the intention-to-treat analyses. Post-hoc sensitivity analyses used data only from participants who completed the educational activities and collapsed the study arms into two categories (education topic and control) for regression modelling [32].

### Qualitative interview analysis

The qualitative data were coded thematically under the domains of the COM-B framework [35, 36]. This framework proposes that human behaviour (B) is best understood through the interaction between capability (C) (physical and psychological), Opportunity (O) (social and physical) and Motivation (M) (automatic and reflective). The COM-B framework is part of the larger Behaviour Change Wheel (BCW) framework [35, 36], and associated Theoretical Domains Framework (TDF) [37, 38], and is useful in guiding an understanding of human behaviour through the interactions between capability, opportunity, and motivation. The COM-B framework has previously been used to explore mechanisms of behaviour change in general practice settings and so was considered appropriate to use as a framework in our study. COM-B has been used for assessing quality improvement interventions such as behaviour change in documented assessment during routine consultations [39] and behaviour change through learning in practice for pharmacists in primary care settings [40]. It has also been used to help understand barriers and facilitators to chlamydia testing in general practice [41]. As pre- and post-intervention qualitative interviews were conducted with the same participants, we were able to examine the interactions of the components in the COM-B framework and to look at the resulting changes in behaviour post education intervention towards using MHR and rational prescribing and test ordering [32].

## Results

### Educational questionnaire results

Of the 106 participants enrolled into the larger study, baseline questionnaires were returned by 60 participants and post-education questionnaires were returned by 37 participants. Thirty-nine baseline participants and all 37 post-education questionnaire participants completed the education activities. The sex and age distribution of participants were broadly similar to the study sample as a whole [24]. See Table 1.

**Table 1:** Respondent characteristics

	**Baseline** *N* = 60	**End** *N* = 37
**Sex**		
Male	38 (64%)	25 (69%)
Female	21 (36%)	11 (31%)
Unknown	1	1
**Age in years (mean)**	49 (41, 60)	51 (40, 60)
Unknown	1	1
**Education completed**		
Complete	39 (65%)	37 (100%)
Not complete	21 (35%)	0 (0%)
**Study arm**		
Imaging	18 (30%)	14 (38%)
Pathology	20 (33%)	12 (32%)
Prescribing	22 (37%)	11 (30%)

The proportion of participants who rated themselves as ‘Extremely confident’ in their use of MHR, changed from 8.3% at baseline to 35% post-education. Participants who reported using MHR over 30 times in the previous three months also changed, from 1.7% at baseline to 8.1% post-education. Regression models also demonstrated significant improvements overall in confidence (*p* < 0.001) and self-reported MHR use (*p* < 0.001) [24]. See Table 2 for MHR items responses.

**Table 2:** MHR item responses

	**Baseline** *N* = 60	**End** *N* = 37
**MHR confidence**		
1—not confident	14 (23%)	0 (0%)
2	14 (23%)	0 (0%)
3	8 (13%)	4 (11%)
4	19 (32%)	20 (54%)
5—extremely confident	5 (8.3%)	13 (35%)
**MHR use**		
0 times	16 (27%)	0 (0%)
1–10 times	32 (53%)	16 (43%)
11–20 times	7 (12%)	13 (35%)
21–30 times	4 (6.7%)	5 (14%)
31 + times	1 (1.7%)	3 (8.1%)
**Appropriate MHR record**		
Event Summary	5 (8.3%)	3 (8.1%)
Medicines View Summary	20 (33%)	11 (30%)
Shared Health Summary	35 (58%)	23 (62%)

While the proportions of respondents who rated themselves as confident or very confident in deprescribing increased by the end of the trial across the cohort overall (*p* < 0.01), there were no significant differences in the changes between arms. There were no significant changes between arms in reported frequency of deprescribing discussions or actions. See Table 3.

**Table 3:** Prescribing question responses for cohort overall

	**Prescribing arm**		**Pathology arm**		**Imaging arm**	
	**Baseline** *N* = 22	**End** *N* = 11	**Baseline** *N* = 20	**End** *N* = 12	**Baseline** *N* = 18	**End** *N* = 14
**Deprescribing confidence**						
1—not confident	2 (9.1%)	0 (0.0%)	0 (0.0%)	0 (0.0%)	1 (5.6%)	0 (0.0%)
2	4 (18.2%)	0 (0.0%)	4 (20.0%)	0 (0.0%)	1 (5.6%)	0 (0.0%)
3	10 (45.5%)	1 (9.1%)	9 (45.0%)	4 (33.3%)	9 (50.0%)	5 (35.7%)
4	5 (22.7%)	7 (63.6%)	5 (25.0%)	6 (50.0%)	7 (38.8%)	6 (42.9%)
5—extremely confident	1 (4.5%)	3 (27.3%)	2 (10.0%)	2(16.7%)	0 (0.0%)	3 (21.4%)
**Deprescribing discussions**						
0 times	0 (0.0%)	0 (0.0%)	1 (5.0%)	0 (0.0%)	1 (5.6%)	0 (0.0%)
1–5 times	10 (45.4%)	0 (0.0%)	6 (30.0%)	3 (25.0%)	5 (27.8%)	2 (14.3%)
6–10 times	8 (36.4%)	5 (45.4%)	8 (40.0%)	3 (25.0%)	7 (38.9%)	7 (50.0%)
11–15 times	2 (9.1%)	3 (27.3%)	4 (20.0%)	3 (25.0%)	3 (16.6%)	3 (21.4%)
16 + times	2 (9.1%)	3 (27.3%)	1 (5.0%)	3 (25.0%)	2 (11.1%)	2 (14.3%)
**Deprescribing frequency**						
0 times	2 (9.1%)	0 (0.0%)	0 (0.0%)	0 (0.0%)	0 (0.0%)	0 (0.0%)
1–5 times	8 (36.4%)	2 (18.2%)	11 (55.0%)	2 (16.7%)	7 (38.9%)	3 (21.4%)
6–10 times	7 (31.8%)	5 (45.4%)	4 (20.0%)	7 (58.3%)	4 (22.2%)	6 (42.9%)
11–15 times	3 (13.6%)	3 (27.3%)	2 (10.0%)	0 (0.0%)	5 (27.8%)	2 (14.3%)
16 + times	2 (9.1%)	1 (9.1%)	3 (15.0%)	3 (25.0%)	2 (11.1%)	30 (21.4%)

In the cohort overall, participants reported a significant increase in confidence in evidence-based pathology ordering (*p* < 0.01), with a significant increase in the pathology education arm compared with the prescribing education arm (*p* = 0.03). Self-assessed frequency of review of pathology test ordering also increased significantly in the cohort overall by the end of the trial (*p* < 0.001), with the changes significantly greater in the pathology education arm compared with the other arms (pathology vs. imaging *p* < 0.01; pathology vs prescribing < 0.001). See Table 4.

**Table 4:** Pathology question responses for cohort overall

	**Prescribing arm**		**Pathology arm**		**Imaging arm**	
	**Baseline** *N* = 21	**End** *N* = 6	**Baseline** *N* = 16	**End** *N* = 9	**Baseline** *N* = 18	**End** *N* = 11
**Confidence in evidence based pathology ordering**						
1—not confident	0 (0.0%)	0 (0.0%)	0 (0.0%)	0 (0.0%)	0 (0.0%)	0 (0.0%)
2	3 (14.3%)	1 (16.7%)	2 (12.5%)	0 (0.0%)	2 (11.2%)	0 (0.0%)
3	9 (42.9%)	3 (50.0%)	7 (43.8%)	1 (11.1%)	8 (44.4%)	4 (36.4%)
4	8 (38.0%)	2 (33.3%)	7 (43.8%)	6 (66.7%)	8 (44.4%)	6 (54.5%)
5—extremely confident	1 (4.8%)	0 (0.0%)	0 (0.0%)	2 (22.2%)	0 (0.0%)	1 (9.1%)
**Pathology review frequency**						
0 times	6 (28.6%)	3 (50.0%)	2 (12.5%)	0 (0.0%)	1 (5.6%)	1 (9.1%)
1–5 times	8 (38.1%)	3 (50.0%)	9 (56.3%)	5 (55.6%)	10 (55.5%)	5 (45.4%)
6–10 times	5 (23.8%)	0 (0.0%)	2 (12.5%)	2 (22.2%)	5 (27.7%)	3 (27.3%)
11–15 times	2 (9.5%)	0 (0.0%)	2 (12.5%)	1 (11.1%)	1 (5.6%)	1 (9.1%)
16 + times	0 (0.0%)	0 (0.0%)	1 (6.2%)	1 (11.1%)	1 (5.6%)	1 (9.1%)
**Pathology regimen change frequency**						
0 times	1 (4.8%)	0 (0.0%)	0 (0.0%)	1 (11.1%)	0 (0.0%)	0 (0.0%)
1–5 times	6 (28.6%)	3 (50.0%)	5 (31.3%)	1 (11.1%)	6 (33.3%)	7 (63.6%)
6–10 times	10 (47.6%)	0 (0.0%)	1 (6.2%)	2 (22.2%)	7 (38.9%)	2 (18.2%)
11–15 times	0 (0.0%)	1 (16.7%)	4 (25.0%)	2 (22.2%)	1 (5.6%)	1 (9.1%)
16 + times	4 (19.0%)	2 (33.3%)	6 (37.5%)	3 (33.4%)	4 (22.2%)	1 (9.1%)

Across the cohort overall, confidence in evidence-based imaging ordering for low back pain significantly increased (*p* < 0.001), as did self-reported frequency of discussions for reasons for not ordering imaging (*p* = 0.049). The increase in confidence was significantly greater in the imaging arm compared with the pathology and prescribing arms (*p* < 0.001). There were no other significant changes in the imaging responses. See Table 5.

**Table 5:** Imaging question responses for cohort overall

	**Prescribing arm**		**Pathology arm**		**Imaging arm**	
	**Baseline** *N* = 21	**End** *N* = 6	**Baseline** *N* = 16	**End** *N* = 9	**Baseline** *N* = 18	**End** *N* = 11
**Confidence in evidence based imaging**						
1—not confident	0 (0.0%)	1 (16.7%)	0 (0.0%)	0 (0.0%)	0 (0.0%)	0 (0.0%)
2	3 (14.3%)	0 (0.0%)	2 (12.5%)	3 (33.3%)	2 (11.1%)	0 (0.0%)
3	10 (47.6%)	1 (16.7%)	8 (50.0%)	3 (33.3%)	7 (38.9%)	3 (27.3%)
4	8 (38.1%)	4 (66.6%)	6 (37.5%)	2 (22.3%)	8 (44.4%)	5 (45.4%)
5—extremely confident	0 (0.0%)	0 (0.0%)	0 (0.0%)	1 (11.1%)	1 (5.6%)	3 (27.3%)
**Frequency guideline use in back pain imaging**						
0 times	7 (33.3%)	2 (33.3%)	2 (12.5%)	4 (44.5%)	5 (27.8%)	0 (0.0%)
1–5 times	8 (38.1%)	3 (50.0%)	11 (68.8%)	3 (33.3%)	8 (44.4%)	5 (45.4%)
6–10 times	5 (23.8%)	0 (0.0%)	3 (18.7%)	1 (11.1%)	3 (16.7%)	3 (27.3%)
11–15 times	1 (4.8%)	1 (16.7%)	0 (0.0%)	0 (0.0%)	2 (11.1%)	3 (27.3%)
16 + times	0 (0.0%)	0 (0.0%)	0 (0.0%)	1 (11.1%)	0 (0.0%)	0.0 (0.0%)
**Frequency discussed why not ordering imaging**						
0 times	0 (0.0%)	0 (0.0%)	0 (0.0%)	0 (0.0%)	0 (0.0%)	0 (0.0%)
1–5 times	12 (57.1%)	1 (16.7%)	9 (56.3%)	2 (22.2%)	9 (50.0%)	3 (27.3%)
6–10 times	5 (23.8%)	3 (49.9%)	5 (31.3%)	6 (66.7%)	4 (22.2%)	2 (18.2%)
11–15 times	1 (4.8%)	1 (16.7%)	1 (6.2%)	0 (0.0%)	2 (11.1%)	4 (36.3%)
16 + times	3 (14.3%)	1 (16.7%)	1 (6.2%)	1 (11.1%)	3 (16.7%)	2 (18.2%)

The results of the sensitivity analyses did not substantively differ from those of the primary analysis models.

### Qualitative results

Nineteen GPs (seven females and twelve males) completed interviews both pre- and post-intervention (a total of 38 interviews). The participant sample in Table 6 shows variation by sex and age of the GPs, and size, remoteness [33] and socio-economic indexes for areas (SEIFA) for practices [34].

**Table 6:** Qualitative interview participant sample

		**Number of participants**
**TRIAL ARM**	**Prescribing**	7
	**Pathology**	7
	**Imaging**	5
**Sex**	Female	7
	Male	12
**Age**	≤ 45	8
	≥ 46	11
**Practice size**	≤ 5	11
	≥ 6	8
**RRMA**	RA1-2	10
	RA3-5	8
	RA6-7	1
**SEIFA**	1–5	12
	6–10	7

RRMA is the Rural, Remote and Metropolitan classification system with RA1-2 classified as Metropolitan, RA 3–4 Rural and RA 6–7 Remote

The SEIFA decile is based on the Index of Relative Socio-economic Disadvantage and describes

the decile ranking of the participating practices, with a decile of 6–10 indicating practices of least

disadvantage and 1–5 of most disadvantage

Table 7 presents the themes under the headings of Capability, Opportunity and Motivation from the COM-B framework [35]. Findings from the interview data on perceived behaviour change are presented below under these headings.

**Table 7:** Capability, opportunity and motivation subthemes

Capability	Opportunity	Motivation
Increased engagement with and knowledge of MHR	Quarantined time to learn about MHR in a time-poor environment	Motivation to learn more about using MHR
Increased knowledge about rational prescribing and test ordering post-intervention	Opportunity to learn more about rational prescribing and test ordering	Motivation to engage more with MHR and to incorporate its use into clinical practice
		Motivation to reflect on deprescribing and test ordering
		Greater agency in refusing low value testing

### Capability

Participants experienced varying degrees of engagement with MHR and for many, there were gaps in their knowledge about how to use it. Post-intervention participants perceived their MHR capability and their rational prescribing and test ordering capability had increased.

#### Increased engagement with and knowledge of MHR

Pre-intervention, most participants perceived that their MHR capability was lacking, whereas post-intervention they reported an increase in their MHR capability, particularly in learning how to use the program, accessing test results and creating shared health summaries. Participants GP21 and GP8 illustrate this perceived increase in MHR capability from pre- to post-intervention:

My sense is that there probably is a bit more stuff in there [MHR system] that I haven’t really managed. (GP21 pre, pathology, female)

I hadn’t realised that you could increasingly get the hospital results from it [MHR system] to be able to see the specialist investigations that they’ve done in the hospital is amazing. (GP21 post, pathology, female)

Sometimes it's a bit tricky when they say they have a health record done elsewhere... I don't know whether I'm actually not doing it properly... I don't know. (GP8 pre, prescribing, male)

I didn’t actually realise that it could be quite comprehensive, like it’s not just the health summary, it contains a lot of information from the hospital as well…. Now I use it a lot for patients who recently have been to hospital, and if you wait three or four days a lot of the results will be uploaded. (GP8 post, prescribing, male)

Likewise, GPs who were not actively engaged with MHR prior to the intervention changed their MHR usage behaviour post-intervention. Their increase in MHR skills, ability and knowledge both enabled and encouraged them to use MHR more:

I wasn’t really doing much uploading of health summaries and things for patients because I just wasn’t sure how to do it and I didn’t want to accidentally upload the wrong thing… So, I think I did learn a lot from the experience. (GP7 post, imaging, female)

I think, became a bit more confident and familiar with the My Health Record, obviously. As a result, I’ve used it more than I would have otherwise. (GP4 post, prescribing, male)

However, there were a few participants post-intervention that perceived they still had knowledge gaps in regard to feeling confident using MHR:

I still don’t feel confident of what should I be uploading to the My Health Record. How do I make the most of the resources that are there? I guess, I’m just a bit unfamiliar with the technology and really how to get the most out of it. (GP16 post, imaging, female)

#### Increased knowledge about rational prescribing and test ordering post-intervention

Among most participants, there appeared to be a lack of knowledge regarding rational prescribing and test ordering in the context of MHR. However, post-intervention, the majority of participants perceived an increase in knowledge and a change of behaviour in rational prescribing and test ordering. This is illustrated below from a participant in the prescribing arm:

I don’t know anything about that, I’m sorry. That’s an area I have to gain knowledge on [rational prescribing and test ordering]. (GP23 pre, prescribing, female)

I was put into the prescribing arm and through the webinar I learnt about the resources that could be used for deprescribing and how it could be done safely and involving patients in making that decision about reducing the pill burden… I never knew about some of the resources that they had given us through the course about each medication that could be safely deprescribed (GP23 post, prescribing, female)

### Opportunity

The education intervention gave participants the opportunity to learn more about how to use MHR in the context of rational prescribing and test ordering.

#### Quarantined time to learn about MHR in a time-poor environment

Many participants in the pre-intervention interviews perceived that time constraints were a major barrier to social opportunities in using MHR:

I have used it a little bit in that we have been trying to get some of our patients on board, although it normally falls off the bottom of the to do list. So I'm finding that, although I intend to sign people up, actually getting to the point where I'm signing them up and activating the file isn't always happening… at the end it just fell off the bottom of the learning list. (GP16 pre, imaging, female)

It's quite difficult because it's several extra steps that we need to do in an already time poor environment. (GP14 pre, pathology, female)

As well as being time-poor, participants noted that MHR was a non-user friendly system, and perceived it to be “slow” and “clunky” to use:

Look, it’s really slow to upload, so it’s just a clunky thing... So I guess in a way, I’m hoping that it [the education intervention] then gives me almost a bit of quarantined time to really have a better look at it and see what else is there and see how else I can be using it. (GP21 pre, pathology, female)

The education intervention was seen by some as an opportunity for “quarantined time” to focus on learning more about MHR:

I’m just thinking about looking at it now [MHR], where I didn’t even bother with most of my patients… (GP16 post, imaging, female)

Many GPs also perceived that the education intervention may have a positive impact on their work efficiency and clinical practice:

I’m hoping that through education, I’ll be a better proponent for the tool, and that I’ll use the tool more effectively… So any means by which I could increase efficiency with utilising that tool will go on to better serve me. (GP25 pre, prescribing, male)

GP12 in her pre-intervention interview, thought the education would provide the opportunity for her to become more efficient and describes her efforts at using MHR post-intervention:

The education. I think it might make me more efficient. I mean, in GP practice, you're doing a whole lot of things at the same time, and so to upload at the same time as doing everything else, if you're more slick at doing it because you've practiced and been educated, I think it's better. (GP12 pre, prescribing, female)

I know that our practice is very guilty of this, that things get left on for years when a patient is no longer using them, and it [the education intervention] would make me go through and check everything, make sure that what was on their medication list was current, and then I would upload that on to the eHealth record as the most recent summary. So I guess, yeah, for that reason it just enthused me to do that perhaps a bit more than I would otherwise. (GP12 post, prescribing, female)

#### Opportunity to learn more about rational prescribing and test ordering

Pre-intervention, several participants perceived that the education intervention may impact their prescribing and test ordering behavior but were not sure about the processes involved. Post-intervention many participants gave examples of how the intervention provided an opportunity to learn more about rational prescribing and test ordering, such as GP21 from the pathology arm:

Part of me that’s a bit pleased that I’m in the pathology arm because I’m not quite sure how it will change what I do with that. (GP21 pre, pathology, female)

I wasn’t expecting but found (the education intervention) very useful just that review of which tests are not overly helpful and how often we order them. So, I wasn’t expecting that but it was good to sort of re-cap that. (GP21 post, pathology, female)

One participant described before and after the education the opportunity that the education gave them in terms of deprescribing:

With prescription, that is an arm in particular that I have not utilised before, so this will be quite interesting to see the impact… I really have definitely been under-utilising it… It doesn’t inform my current practice, My Health Record, and it probably should and hopefully post-education there will be a change. (GP25 pre, prescribing, male)

Prior to going on to the study, I was aware of the need for deprescribing… it helped to consolidate that fact, but more importantly, it gave me a structure… an arsenal of resources… it's given me a starting block… and the information on how to better arm that conversation between me and the patient. (GP25 post, prescribing, male)

### Motivation

The education reinforced learning about MHR and rational prescribing and test ordering and motivated changes in behaviour.

#### Motivation to learn more about using MHR

A main motivator GPs noted for participating in the study was to learn how to use MHR, and/or to improve their use of MHR. The following participant explains in their pre- and post-interviews about being motivated to learn more about MHR:

To be quite frank with you, anything about My Health Record would be good, because at the moment, I understand its benefits. I kind of have used it, but not very much… So learning how to use it in the most useful way for when I do... (GP7 pre, imaging, female)

So, learning how to utilise My Health Record to be a more efficient GP was kind of my aim and I think also allowing patients to be on their front foot, so learning how to upload…I can see the benefit of having the uploaded view of the patient because there’s so much that you can access. So, I think I did learn a lot from the experience. (GP7 post, imaging, female)

#### Motivation to engage more with MHR and to incorporate its use into clinical practice

Many participants reported that they were motivated to use MHR more post-intervention, particularly as the education had provided the opportunity to increase capability and be more confident in using it. There are many examples of automatic motivation where the education reinforced learning and motivated participants to engage with MHR more and to incorporate it into their practice routines.

Coming out of the [education] program, I feel really guilty that I never looked [at MHR]… but now actually coming back, yeah it has actually changed the way I look at things and doing more searching for online results, which I did this morning for a patient. (GP3 post, pathology, male)

I've made some changes as in I’m getting used to the process of using My Health Record for a patient. But it will become like a habit, the same as looking at the patient’s medications when I am seeing new patients, patients who come back or a new patient, and do all the medications. (GP19 post, pathology, female)

There are also examples of reflective motivation where some participants were motivated by intentions to use MHR in clinical practice for the benefit of their patients and as a way of reducing unnecessary tests:

It seemed to be a way of getting information to us as how to use things like My Health Record, in line with imaging for me… the ways to have guidelines, to appropriate imaging and the like, where to look for those sorts of things, and that’s – that’s been extremely useful. (GP10 post, imaging, male)

I suppose I was aware of it in a sense but not using it, so it was good to learn a bit more about it I suppose in a practical sense of how to use it for the benefit of my patients. (GP6 post, pathology, male)

#### Motivation to critically reflect on rational prescribing and test ordering

It was perceived that the education motivated participants to engage in critical thinking about rational prescribing and test ordering; “For me it was just not routinely ordering stuff because that’s what we always do” (GP21 post, pathology, female):

It’s very easy to lull into “this is my shortcut for this, and therefore I’ll just click that and it will order all these tests”. Whereas trying to think more critically about a patient, I’ve been trying to do that a lot more… I am sort of questioning, do I really need to do this test? (GP14 post, pathology, female)

One participant in the imaging arm described pre- and post-intervention how the education motivated them to reflect on their imaging practice:

Look, I think it will be useful to reflect on what my referral to imaging practice is because I know from NPS audits that I order some things more than my peers and other things less. So I guess it would be interesting to actually look at that and reflect on it and to feel that the things I'm referring for that are best practice. (GP16 pre, imaging, female)

I’ve started to think more about what I’m ordering. I’ve started to say “no” more to some patients when I feel the test isn’t really clinical indicated, and feel more confident with that, saying “no” rather than just ordering a test just to make sure, because the patients pushing for it. (GP16 post, imaging, female)

Participants also noted that the format of the education, motivated them to reflect on their practice and prompted them to change their prescribing and test ordering behaviour:

It was actually quite helpful from a reflection point of view, and I think that for me at least, that was what sort of changed my behaviour, having to reflect back on the cases that I’ve done… having to submit those cases, pushing me into doing, having that first step in, and changing how I order tests. (GP14 post, pathology, female)

#### Greater agency in refusing low value test ordering

Participants across the three arms gave examples of the education providing them the opportunity for greater agency to change their prescribing and test ordering behaviour post-intervention. Participants in the prescribing arm gave examples of how they have begun deprescribing and how the education has given them the tools to do so. Participants in the imaging and pathology arms noted how the education has given them the “confidence” to say no to tests when they felt it was justified. The following two participants give examples of their behaviour change pre/post-intervention, with GP21 saying that the education has given them “more agency” and GP11 saying they now have the “courage” to say no to a patient that asks for a low value or unnecessary test:

Part of me that’s a bit pleased that I’m in the pathology arm because I’m not quite sure how it will change what I do with that. (GP21 pre, pathology, female)

It just gave me a bit more agency to be able to say to people, “Look actually, we’ve checked the TSH every 12 or 18 months for the last four or five years, and it’s always been normal, and this is what the evidence tells us and if we have several readings that are normal, that really the chance of there being anything abnormal without any symptoms developing, is really incredibly low, and I don’t think we need to check it again”. (GP21 post, pathology, female)

I'm in the pathology arm. I'm hoping that it highlights what I'm ordering too much of, or I'm not ordering enough of or how I'm not comparing with my peers. (GP11 pre, pathology, male)

It was really useful… and absolutely applicable for general practice… I’ve been a GP 30 years, I do try and think about what I order with my pathology tests and this has really brought it into focus for me and I’m far more comfortable now not ordering tests… I’ve got a 98-year-old guy who keeps insisting that we measure his PSA. I’ve now got the courage to say to him, “Mate, are you going to live another 7 years?” I’m not going to do it this time. (GP11 post, pathology, male)

This reflective motivation also led some participants to opportunistically apply concepts learned in their study arm and apply it in another; for example, a participant in the pathology arm was motivated to change their prescribing behaviour:

It will become like a habit, the same as looking at the patient’s medications when I am seeing new patients, patients who come back or a new patient, and do all the medications. (GP19 post, pathology)

## Discussion

The self-report findings for our study indicate behaviour change as a consequence of the education intervention. Overall, our findings strongly suggest that the education intervention had a positive impact on improving behaviours and attitudes towards MHR use, and towards rational medication prescribing and rational ordering of pathology and imaging.

The questionnaire results showed that there was a significant increase in confidence using MHR, and a corresponding increase in the self-reported frequency of the use of MHR [24]. There were similar all-cohort changes pre-post-intervention in confidence in frequency of review of pathology ordering regimens and evidence-based imaging and in deprescribing. There were significant between-arm improvements in the pathology arm in self-assessed frequency of review of pathology ordering regimens, and in the imaging arm in confidence in evidence-based imaging. There is some indication that there was an overlap of the effects of the educational interventions across arms [32].

The qualitative findings suggest that the education package increased GPs’ awareness, knowledge, capability with, and use of MHR. The COM-B framework was an effective way to explore the mechanisms of behaviour change that were brought about through the education intervention. Participants perceived more frequently utilising MHR, particularly in aiding informational continuity of care, and in helping to make better clinical decisions in the management of the health of their patients. The education also gave participants the tools to increase capability in rational prescribing and test ordering. Participants perceived an increase in using MHR as a tool in reducing unnecessary tests. Participants also perceived an increase in their confidence in deprescribing, and in rational pathology and imaging ordering. Our primary findings reported elsewhere [30] showed statistical significance in changes in pathology costs in the cohort that completed the education. This highlights the potential that educational interventions can have in enabling cost savings and quality improvement in the health system.

Comparison of our findings with those of other studies confirms the effectiveness of multifaceted or multicomponent intervention approaches [1, 3]. Similar to previous studies, our participants perceived that the interactive education which incorporated guidelines and drug usage advice, clinical audit activity and reflective practice, were a motivator to change their prescribing and test ordering behaviours [17–20]. Challenges such as time constraints and the need for software to be more user friendly were issues we found that have also been raised in previous research [42]. Also similar to other studies, our findings showed that participants perceived there would be potential benefit from being able to compare ordering and prescribing statistics with their peers in future research [17, 20].

### Limitations

There were several limitations to the study. The education session completion rate was suboptimal, likely due to the intervention taking place during the Covid-19 pandemic, which impacted the overall effect of interventions in the cohort. The 19 participants that consented to be interviewed both pre- and post-intervention were those who had completed the education or intended on completing it, therefore, it was not possible to include perceptions and experiences from participants who did not intend to complete the education. Likewise, the 37 participants included in the post-education questionnaire analysis were participants that had completed all the education activities. This may be one reason why this paper reports on accounts of positive behavioural change across the three arms of the study, whereas the wider study did not demonstrate statistical change in the intention-to treat cohort. Furthermore, the education intervention did not include ancillary or follow-up reinforcement activities, such as feedback on performance which would have provided further information about the sustained impact of the educational interventional on prescribing and test ordering behaviours.

### Future research

Future directions for development and evaluation of scalable quality improvement activities incorporating MHR and rational prescribing and test ordering include exploring ways to improve the user friendliness of MHR and the education modules as well as incorporating reinforcing activities such as real-time feedback of performance. We hypothesise that in this study, principles and skills learned in rational prescribing and test ordering were transferrable across topic areas, resulting in overall cohort changes in confidence. This has implications for future interventions and research in quality use of tests and medicines, as wider system impact may be gained from leveraging ‘transferrable concepts’ in tandem with practice in specific topics.

## Conclusion

This study demonstrated mechanisms by which an education intervention had effect, through increasing GP capability, providing opportunity and enhancing motivation in MHR knowledge and usage, as well as rational prescribing and test ordering behaviour. It appeared that concepts and skills learned in one domain were transferrable to other domains, across prescribing and test ordering. This has implications for the design of medical education in quality improvement and the potential for broadening education impact.

### Supplementary Information

**Additional file 1.** Pre- and post-interview guides.

**Additional file 2.** Pre- and post-education questionnaires.

## Data Availability

To protect the privacy and confidentiality of individual GPs, primary data is not made publicly available. Contact Dr Christine Metusela (metusela@uow.edu.au) for matters relating to data availability.

## Abbreviations

ADHA: Australian Digital Health Agency
EHR: Electronic Health Record
GP: General practitioner
MBS: Medicare Benefits Schedule
MHR: My Health Record
PBS: Pharmaceutical Benefits Scheme
RACGP: Royal Australian College of General Practitioners
RRMA: Rural, Remote and Metropolitan Area
SEIFA: Socio-economic index for areas
UOW: University of Wollongong
