# Supplementary material for: CHIME-GP trial of online education for prescribing, pathology and imaging ordering in general practice – how did it bring about behaviour change?
Source: BMC Health Serv Res. 2023 Dec 2;23:1346. doi: 10.1186/s12913-023-10374-1 (PMC10693689; doi:10.1186/s12913-023-10374-1)
Supplement: Supplementary file 1 — Additional file 1. Pre- and post-interview guides. [file 12913_2023_10374_MOESM1_ESM.pdf]

Additional File 1: Pre- and post-interview guides

Pre-intervention interview

This evaluation is reviewing the Medcast's education intervention using MyHR.

What are you hoping to achieve/learn? How are you hoping to achieve that?

Prompts

i. Learn how to use MyHR (skills)
ii. Improve clinical practice
iii. Learn about de-prescribing/pathology/radiology
iv. Improve use of MyHR
v. CPD

Can you tell me about your current engagement with MyHR?

Prompts

vi. No engagement- I'm interested to understand why you haven't engaged with MyHR?
vii. Attitude? Patient and GP.
viii. Is it useful?
ix. Acceptability?
x. Sustainability?
xi. Concerns?
xii. Benefits? Shared records across healthcare providers

How do you think this intervention will change how you currently use MyHR?

Prompts

i. Impact on clinical practice/work efficiency
ii. Impact on ordering of pathology/radiology
iii. Impact on prescribing
iv. It won't change anything - can you explain why you think this is the case?

This education intervention will involve several learning modules including case based webinars , an online learning module and an audit of five patients as a way to apply the skills learnt in the other workshops (NB: audit only for interviewees participating in prescribing and pathology intervention)

Can you tell me what you think of this format to support CPD?

Prompts

i. Usefulness?
ii. Acceptability?
iii. Sustainable way for GP CPD?

a. What would you see as being barriers to this format?
b. What are the benefits of this format?

Thank you very much for your time today. If you have any queries about this discussion afterwards, please do not hesitate to contact the research team on the numbers supplied on the participant information sheet.

Post-intervention interview

In the baseline interview we talked about what you thought you would achieve/learn in this education program and your current engagement with MyHR.

Now that you have participated in the program, was it what you expected? What do you feel (if anything) you have learnt? Will it change your practice in any way?

Prompts

i. Learn how to use MyHR (skills)
ii. Improve clinical practice
iii. Prescribing/pathology/radiology ordering
iv. Use of MyHR
v. CPD purposes

Has this intervention changed how you use MyHR?

b. Do you plan to make or have you already made any changes to your practice?
c. Did the education program allow you to identify how you might introduce these changes?

Prompts

i. Do you think will have an Impact on clinical practice/work efficiency
ii. Do you think will have an Impact on Ordering of pathology/radiology
iii. Do you think will have an Impact on Prescribing
iv. It hasn't- can you explain why you think this is the case?

This education intervention involved several learning modules including case based webinars , an online learning module and an audit of five patients as a way to apply the skills learnt in the other workshops

Can you tell me what you thought of this format to support CPD?

Prompts

i. Usefulness?
ii. Acceptability?
iii. Sustainable way for GP CPD? Or learn about MyHR?

a. What were the barriers to this format?
b. What were the benefits of this format?

Thank you very much for your time today. If you have any queries about this discussion afterwards, please do not hesitate to contact the research team on the numbers supplied on the participant information sheet.
