# Supplementary material for: CHIME-GP trial of online education for prescribing, pathology and imaging ordering in general practice – how did it bring about behaviour change?
Source: BMC Health Serv Res. 2023 Dec 2;23:1346. doi: 10.1186/s12913-023-10374-1 (PMC10693689; doi:10.1186/s12913-023-10374-1)
Supplement: Supplementary file 2 — Additional file 2. Pre- and post-education questionnaires. [file 12913_2023_10374_MOESM2_ESM.pdf]

Medcast

Rational Prescribing: Pre / Post Training Quiz

1. Please indicate your level of confidence using the medicines information view of My Health Record (MHR) with patients as part of your clinical practice.
    a. 1 - not confident
    b. 2
    c. 3
    d. 4
    e. 5 - extremely confident

2. In the last 3 months, how many times have you used MHR to assist with medication information during patient consultations?
    a. 0 times
    b. 1-10 times
    c. 11-20 times
    d. 21-30 times
    e. 31+ times

3. Please indicate your level of confidence in your approach to deprescribing medications in the elderly?
    a. 1 - not confident, no formal approach
    b. 2
    c. 3
    d. 4
    e. 5 - extremely confident

4. In the last 3 months, how often have you opportunistically discussed deprescribing with a patient?
    a. 0 times
    b. 1-5 times
    c. 6-10 times
    d. 11-15 times
    e. 16+ times

5. In the last 3 months, how often have you stopped a medication that you deemed no longer necessary?
    a. 0 times
    b. 1-5 times
    c. 6-10 times
    d. 11-15 times

e. 16+ times

6. Do you have a particular approach to deprescribing? Y/N

7. If you have answered yes, please describe your approach. (free text)

8. What percentage of patients are inappropriately prescribed medicines?

a. Up to 10%
b. Up to 25%
c. Up to 40% - correct
d. Up to 60%
e. Up to 75%

9. You review a patient who is on holiday and prescribe a new medication. They are not a regular patient of yours. Which of the following would be most appropriate to upload to their MHR?

a. Event Summary - correct
b. Shared Health Summary
c. Medicines View Summary
d. Discharge Summary
e. Specialist Letter

10. You complete a 75 year health assessment on a regular patient of yours with multiple comorbidities, and deprescribe a medication. Which of the following would be most appropriate to upload to their MHR?

a. Event Summary
b. Shared Health Summary - correct
c. Medicines View Summary
d. Discharge Summary
e. Specialist Letter

11. For which medication classes is there good evidence to consider deprescribing? *(Answer: PPIs, opiates, BZD, ICS)*

a.
b.
c.
d.

Question in the Post-survey only

Please give examples of when you have desprescribed a patient's regular medication. Include a brief description of the patient presentation, medication/s that was deprescribed and rationale.

a. Example 1 (free text)

b. Example 2 (free text)

c. Example 3 (free text)

d. Example 4 (free text)

Rational Prescribing Case Study

Reginald Webster, aged 75 years, presents to your General Practice as he has been recalled for a Health Assessment for Older Persons ("75-Year-Old Health Check").

Past medical History

- Asthma - this is well managed and he cannot remember the last time he required PRN salbutamol.
- Gout - taking allopurinol for 3 years with good effect, last episode of gout 18 months ago.
- Hypertension - blood Pressure has been stable at around 125/80 for the past 6 years.
- Ischaemic heart disease - CABG performed 10 years ago but remains symptom free.
- Osteoarthritis bilateral knees - takes PRN analgesia for intermittent pain, has noticed his intake of Voltaren is increasing to on average 50mg per day.
- Reflux
- Type 2 Diabetes - commenced metformin 2 years ago,HbA1c has been consistently within limits for the past 12 months.

Reginald complains that he is having trouble sleeping as he is getting heartburn and pain in his knees as he lies down in bed. On further questioning he has made minimal lifestyle changes. His weight has been steadily increasing and his BMI is now 29 kg/m2. He requests a repeat of his prescriptions.

His current medications include:

- Allopurinol 100mg orally daily for hyperuricaemia
- Atorvastatin 40mg orally mane for hyperlipidaemia
- Esomeprazole 40mg orally daily for gastroesophageal reflux
- Frusemide 20mg orally mane for hypertension
- Metformin 1000mg XR orally mane for Type 2 Diabetes Mellitus
- Panadol Osteo 2 tablets orally tds for osteoarthritis
- Perindopril 5mg orally daily for hypertension
- Seretide Accuhaler 500/50 one puff bd inh for chronic asthma
- Salbutamol 100 ug MA (metered aerosol) orally prn for asthma
- Stemetil 5mg orally prn for vertigo
- Temazepam 10mg orally nocte prn for insomnia
- Panadeine Forte 2 tablets qid orally prn for osteoarthritis
- Voltaren 25mg bd orally prn for osteoarthritis

1. What further history should you obtain before prescribing his usual medications?

2. Does the combination of any of the medications listed above concern you? If yes, please list the potential drug-drug interaction and your concerns (free-text):

3. What medications may be contributing to Reginald's symptoms and why (free-text)?

4. What changes would you make, if any, to each of the following medications?

Medication	Current dose / frequency	Change?<br><br>No /<br>Cease/<br>Change Dose	What changes would you suggest (if any)?	What information are you considering when making these changes?
Allopurinol	100mg daily PO			
Atorvastatin	40mg mane PO			
Esomeprazole	40mg daily PO			
Frusemide	20mg mane PO			
Metformin	1000mg XR mane PO			
Panadol Osteo	2 tablets tds PO			
Perindopril	5mg daily PO			
Seretide Accuhaler	500/50 one puff bd			
Salbutamol	100 ug MA orally prn			
Stemetil	5mg prn PO			
Temazepam	10mg orally nocte prn			
Panadeine Forte	2 qid orally prn			
Voltaren	25mg bd orally prn			

5. Are there any medications not currently prescribed that you would like to commence?

Medcast

Diagnostic Imaging: Pre / Post Training Quiz

1. Please indicate your level of confidence using My Health Record (MHR) with patients to access imaging reports as part of your clinical practice.
    a. 1 - not confident
    b. 2
    c. 3
    d. 4
    e. 5 - extremely confident

2. In the last 3 months, how many times you have used MHR to access previous imaging reports during patient consultations?
    a. 0 times
    b. 1-10 times
    c. 11-20 times
    d. 21-30 times
    e. 31+ times

3. Please indicate your level of confidence in ordering diagnostic imaging according to evidence-based guidelines.
    a. 1 - not confident
    b. 2
    c. 3
    d. 4
    e. 5 - extremely confident

4. In the last 3 months, how often have you referred to a guideline when deciding whether to order imaging for a patient with back pain?
    a. 0 times
    b. 1-5 times
    c. 6-10 times
    d. 11-15 times
    e. 16+ times

5. In the last 3 months, how often have you discussed the rationale for not ordering or declining imaging with a patient?
    a. 0 times
    b. 1-5 times
    c. 6-10 times
    d. 11-15 times

e. 16+ times

Diagnostic Imaging - Case Study

Michelle Green, aged 46 years, presents to your general practice with ongoing lower back pain. She is a cleaner by trade and first noticed the pain one week ago when she was squatting and twisting to clean behind a cupboard. She describes onset of lower back pain with shooting pains down her right leg. She took ibuprofen 400mg tds and unpaid sick leave for four days and the pain partially improved. She has since returned to work but finds she is unable to complete her usual duties due to recurrence of pain. The pain makes falling asleep difficult most nights and is present some mornings. It does not wake her overnight. Michelle tells you the pain radiates down her right leg intermittently, usually brought on by working. She has normal power and feeling in her legs. She does not complain of any bowel or bladder symptoms. She does get some stiffness in her back as the day goes on. Michelle does not report any fevers or weight loss. She has been to the physio twice but does not report much improvement. Michelle has no other significant medical history and has had no previous back injuries or trauma to her back. She is a non-smoker and has 2-3 light beers after work each day.

On examination, Michelle's vital signs are within normal limits and her BMI is 28. On examination of her lumbar spine, flexion and extension are limited to 75% due to pain although lateral flexion and rotation are normal. There is some evidence of paravertebral muscle spasm bilaterally at the L4/5 level but no bony tenderness over the vertebrae. Lower limb tone, power, reflexes and sensation are equal bilaterally. Saddle sensation and tone are normal. The quadrant test is positive for local pain on both sides but a little more so on the right. Straight leg raising is limited to 80 degrees bilaterally due to tight hamstring muscles. The slump test appears negative.

Michelle is extremely worried about money as she has not been able to work to her full capacity since her back injury. She is a single parent and would really like this fixed so she can return to work. She is crying and requests that you "find out what is going on".

You review her My Health Record and see she has not had any previous imaging of her low back.

Would you request?	Yes/No	Please provide your rationale

Lumbosacral spine X-ray		
Lumbosacral spine CT scan		
Lumbosacral spine CT scan + steroid injection		
Lumbosacral spine MRI scan (private)		
Lumbosacral spine MRI scan (Work Cover)		
Other (e.g. bloods - please specify)		
No imaging		

- Please note down any other investigations you would consider ordering?*(free text)*

Rational Use of Pathology: Pre / Post Training Quiz

1. Please indicate your level of confidence using My Health Record (MHR) with patients to access pathology reports as part of your clinical practice.
    a. 1 - not confident
    b. 2
    c. 3
    d. 4
    e. 5 - extremely confident
2. In the last 3 months, how many times you have used MHR to access previous pathology reports during patient consultations?
    a. 0 times
    b. 1-10 times
    c. 11-20 times
    d. 21-30 times
    e. 31+ times
3. Please indicate your level of confidence in ordering pathology tests according to evidence-based guidelines.
    a. 1 - not confident
    b. 2
    c. 3
    d. 4
    e. 5 - extremely confident
4. In the last 3 months, how often have you referred to a guideline when requesting pathology, to ensure your requests are evidence-based?
    a. 0 times
    b. 1-5 times
    c. 6-10 times
    d. 11-15 times
    e. 16+ times
5. In the last 3 months, how often have you made changes to a patient's pathology testing schedule, or recalls and reminders?
    a. 0 times
    b. 1-5 times
    c. 6-10 times

d. 11-15 times
e. 16+ times

6. Are you aware of any commonly requested 'low value' pathology tests? (Y/N)

Question in the Post-survey only (pathology)

10. Please give examples of reductions you have made to patients pathology ordering. Include brief description of patient and tests that were not ordered or removed from a schedule.

a. Example 1 (free text)

b. Example 2 (free text)

c. Example 3 (free text)

d. Example 4 (free text)

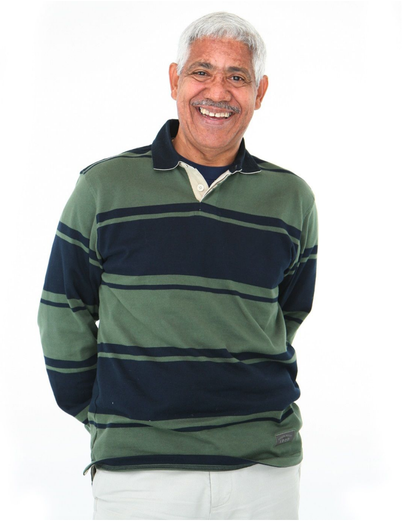

1. Barry White, aged 76 years, presents to your general practice as he has been recalled for Health Assessment for Older Persons ("75-Year-Old Health Check"). Barry is currently well.
Barry's medical history is as follows:

- Gout - one episode/year on average
- Osteoarthritis left knee, mild
- Left inguinal hernia repair - 3 years ago

Family history: nil significant

He is a lifelong non-smoker and has 2-4 units of alcohol each night over the weekend.

Barry is a retired high-school teacher.

On examination Barry's BMI is 27, which has increased from his last check; he has had to buy some new clothes as they were getting tighter. His BP is 140/90 mmHg. The rest of his physical examination is unremarkable.

You note that he had screening for cholesterol and diabetes last year during his health check and both were reported as in the normal range.

Select any pathology investigation, if any, you would request for Barry part of his health assessment today?

Check the investigations you request (Y/N)	If requesting this pathology, please provide your rationale. (free text)
Full blood examination (thought we were taking this out)	
Full blood count	
Urea and electrolytes	
Liver Function Tests	
Thyroid Function Tests	
MSU M/C/S	
Vitamin D	
Please note down any other investigations you would consider ordering?	
